# Supplementary material for: Management and clinical outcomes for patients with gastrointestinal bleeding who decline transfusion
Source: PLoS One. 2023 Aug 25;18(8):e0290351. doi: 10.1371/journal.pone.0290351 (PMC10456126; doi:10.1371/journal.pone.0290351)
Supplement: Attachment — Submitted filename: Response to Reviewers.docx [file pone.0290351.s002.docx]

We thank the Editor and Reviewers for the time and effort taken to suggest edits to improve our manuscript. Below is a point-by-point response addressing these revisions and describing how they were made. All edits in the revised manuscript are shown in underlined, red-colored font.

**Reviewer #1** (No additional comments)

**Reviewer #2:**
Comment- Some types of blood transfusions may be acceptable for each patient
Jehovah’s Witness patients will generally not accept red blood cells, plasma, platelets, or whole blood, other blood components. In some patients, however, may accept autologous blood storage or closed extracorporeal circulation with autotransfusion using a cell saver during the procedure. These options should not be completely ruled out. It is important to confirm the details of precisely which autologous blood transfusions and blood products the patient can accept. This should be added to the DISCUSSION.

Response- The reviewer raises a valid point about the alternative blood products and components that may be acceptable to Jehovah’s Witness patients. In general, these patients do not accept primary blood components such as RBCs, platelets, or plasma. They may be more open to minor blood components like cryoprecipitate, albumin, clotting factors, but this determination is made on an individual basis. Some Jehovah Witness patients are also willing to accept autologous blood salvage and intraoperative hemodilution as these can be performed such that blood remains physically contiguous w/ one’s body. These details have been expounded upon in the revisions of page 14 of the edited manuscript.

[Thank you for correcting and adding references to alternative blood products and components. It is important to confirm these options for each Jehovah's Witness patient. Therefore, if possible, please add to the Discussion, "It is important to confirm the details of which autologous blood transfusions and blood products the patient can accept.

Response- Thank you. This detail has been added to page 15]


Comment- Add to Reference. “Giving HBOCs also require a risk/benefit decision analysis, acknowledging that giving “too little, too late” may not help severely anemic patients, while giving HBOCs for mild or moderate anemia may not be helpful to improve outcomes.” (DISCUSSION, page 12-13). Please add references.

Response- As an experimental therapy, Hemopure is used on a compassionate use basis and requires a risk/benefit decision analysis of factors such as the severity of the patient’s anemia, timely and efficacious administration, and risk factors associated with administration such as increased vasoconstriction and hypertension. Such considerations have been further elaborated in the edits to the revised manuscript on page 14 with additional references (19, 20) cited for deeper exploration of this topic.

[Thank you for the correction. The references are added and will help the reader.]

Comment- Combination of intravenous iron and erythropoietin
In some countries, medical insurance does not cover the use of erythropoietin for anemia due to bleeding. In this study, some or many cases were treated with iron and erythropoietin. Is the increase in Hb greater with iron plus erythropoietin than with iron alone?
If possible, can the authors show the range of increase in Hb for each drug or combination used?

Response- We appreciate the reviewer’s insightful comment about the use of EPO and iron for the correction of anemia and the role that medical insurance might play in the protocol to use either or a combination of both. Patients in our cohort received many different combinations of therapies. As such, it is difficult to isolate the contribution of any one therapy such as of iron, B12, or EPO. Moreover, current literature does not show a consensus concerning whether treatment of anemia is more efficacious with a combination of iron and EPO or iron alone. Some studies demonstrate that there is no difference in the requirement for transfusions when patients are treated with EPO and iron compared to iron alone. Other studies demonstrate that the addition of EPO to iron leads to a greater and faster increase in Hb in anemic patients compared to iron alone. As such, further studies are required to make any decisive conclusions about this, and we have not edited the manuscript based on this comment.

[For the reader, it should be added in the Discussion with references that no conclusion has been reached as to whether the combination of EPO and iron or iron alone is more beneficial.

Response- This is indeed a crucial subject that may inform future protocol. These details have been expounded upon on page 13 with the appropriate references 14-16 added.]
